# Pictorial cigarette pack warnings: a meta-analysis of experimental studies

**DOI:** 10.1136/tobaccocontrol-2014-051978

**Published:** 2015-05-06

**Authors:** Seth M Noar, Marissa G Hall, Diane B Francis, Kurt M Ribisl, Jessica K Pepper, Noel T Brewer

**Affiliations:** 1 School of Journalism and Mass Communication, University of North Carolina at Chapel Hill, Chapel Hill, North Carolina, USA; 2 Lineberger Comprehensive Cancer Center, University of North Carolina at Chapel Hill, Chapel Hill, North Carolina, USA; 3 Department of Health Behavior, Gillings School of Global Public Health, University of North Carolina at Chapel Hill, Chapel Hill, North Carolina, USA

**Keywords:** Packaging and Labelling, Public policy, Global health

## Abstract

**Objective:**

To inform international research and policy, we conducted a meta-analysis of the experimental literature on pictorial cigarette pack warnings.

**Data sources:**

We systematically searched 7 computerised databases in April 2013 using several search terms. We also searched reference lists of relevant articles.

**Study selection:**

We included studies that used an experimental protocol to test cigarette pack warnings and reported data on both pictorial and text-only conditions. 37 studies with data on 48 independent samples (N=33 613) met criteria.

**Data extraction and synthesis:**

Two independent coders coded all study characteristics. Effect sizes were computed from data extracted from study reports and were combined using random effects meta-analytic procedures.

**Results:**

Pictorial warnings were more effective than text-only warnings for 12 of 17 effectiveness outcomes (all p<0.05). Relative to text-only warnings, pictorial warnings (1) attracted and held attention better; (2) garnered stronger cognitive and emotional reactions; (3) elicited more negative pack attitudes and negative smoking attitudes and (4) more effectively increased intentions to not start smoking and to quit smoking. Participants also perceived pictorial warnings as being more effective than text-only warnings across all 8 perceived effectiveness outcomes.

**Conclusions:**

The evidence from this international body of literature supports pictorial cigarette pack warnings as more effective than text-only warnings. Gaps in the literature include a lack of assessment of smoking behaviour and a dearth of theory-based research on how warnings exert their effects.

## Introduction

Tobacco use is the leading cause of preventable death and disease in the world, causing nearly six million deaths each year.^1^ While tobacco product packaging is a key part of marketing efforts to make tobacco use appealing,^2^ ^3^ regulators can use that same packaging to communicate the health risks of tobacco products to consumers.^4^ A pack-a-day smoker potentially sees a cigarette pack an estimated 7300 times per year (20 views/day×365 days/year). Messages on these packs would generate exposure far outweighing exposure from other antitobacco communications, such as mass media campaigns.^5^

The combination of high exposure, nearly universal reach, and very low cost has made pictorial warnings on cigarette packs a core tobacco control strategy globally. The WHO Framework Convention on Tobacco Control (FCTC) calls for the implementation of large warnings on tobacco products.^6^ The treaty's Article 11 specifies that health warnings may include pictures, and subsequent guidelines for implementation state that pictorial warnings are ‘far more effective’ than text-only messages.^6^ By 2015, implementation of pictorial warning policies had occurred in 77 countries and jurisdictions that are home to nearly 50% of the world's population.^7^

As pictorial cigarette pack warnings have proliferated globally, so has research on their impact.^8^ ^9^ Observational studies suggest increased cessation behaviour after the introduction of pictorial warnings,^10^ ^11^ and such studies typically have high external validity. However, isolating the effects of pictorial warnings on smoking behaviour in such studies has proven difficult because governments often introduce the warnings alongside other tobacco control policies.^8^ ^12^ By contrast, experiments can offer strong evidence of the causal impact of pictorial warnings, isolating the effects of warnings on key outcomes. For this reason, experiments are an important tool for studying the effects of pictorial warnings.

### Previous research on pictorial cigarette pack warnings

A large and growing empirical literature has documented the effects of pictorial cigarette pack warnings. Some evidence suggests that pictures and imagery may be more effective than text-only messages at communicating health risks.^13^ ^14^ Compared with text-only warnings, pictorial warnings have been associated with stronger beliefs about the harms of smoking and higher motivation to quit smoking.^10^ ^15–21^ However, while some studies find that smokers and non-smokers rate pictorial warnings as more effective than text-only warnings,^22–26^ other studies have reported conflicting findings.^27–29^ For instance, studies have found that graphic, pictorial warnings result in poorer recall than less graphic or non-graphic warnings,^28^ do not increase youth's expectations to be non-smokers a year later,^29^ have no effect on beliefs about cancer or addiction among non-smoking adolescent boys,^26^ and are effective in lowering smoking intentions for Canadians but not for Americans.^27^

Reviews of the literature on pictorial cigarette pack warnings have taken a variety of approaches. A narrative review by Hammond^8^ suggested that cigarette pack warnings can be effective in promoting smoking cessation, especially when warnings are large, full-colour, and use graphic images. While useful and an important contribution for understanding pictorial warnings, this review did not provide a systematic, quantitative synthesis of pictorial warning effects. A systematic review by Monarrez-Espino *et al*^30^ examined 21 mostly observational studies of the impact of pictorial warnings on reduced smoking, quit attempts and smoking cessation. Monarrez-Espino *et al* found that most of these studies were of poor methodological quality; for this reason, their findings on the impact of pictorial warnings on smoking behaviour were inconclusive. Importantly, this review did not examine many factors that are likely pre-requisites to changes in behaviour, such as attention to warnings, cognitive and emotional reactions to warnings, and changes in beliefs about smoking.

While these recent reviews have summarised portions of the cigarette pack warnings literature,^8^ ^9^ ^30^ no meta-analysis has synthesised the experimental literature on pictorial cigarette pack warnings. To inform international research and public policy, we conducted a meta-analysis of experiments examining the impact of pictorial cigarette pack warnings. Our research question was: across the body of experimental studies, what are the effects of pictorial cigarette pack warnings compared with text warnings?

## Method

### Search strategy

We used a comprehensive search strategy to locate studies relevant to this meta-analysis. The search strategy involved three steps. First, we searched PsycINFO, PubMed, EMBASE, Web of Science, Communication & Mass Media Complete, Business Source Complete, and CINAHL computerised databases in April 2013. We used the following Boolean terms: (cigarette* OR tobacco) AND (warning* OR label* OR pictorial OR graphic OR messag* OR text*). Second, we examined the reference sections of five narrative reviews of cigarette pack warnings.^8^ ^9^ ^31–33^ Third, we examined the reference lists of the final set of articles included in our review. We included all reports that came up in our searches—peer-reviewed journal articles, book chapters, and grey literature (eg, dissertations, publicly available reports)—as long as the full text was available.

To be included, a study had to use an experimental protocol that tested warnings intended for cigarette packs. Studies had to report data on both a pictorial warning condition and a text-only condition. The experimental design could be between subjects (individuals were randomised to different warning label manipulation conditions—eg, text vs pictorial) or within subjects (individuals viewed multiple warning label manipulations). We excluded studies of non-cigarette tobacco products, public service announcements or multicomponent interventions, and warnings embedded in cigarette advertising. We excluded observational studies that asked individuals to report on warnings that they had seen on their own prior to the study. Finally, articles reporting the studies had to be available in English.

**Figure 1:**
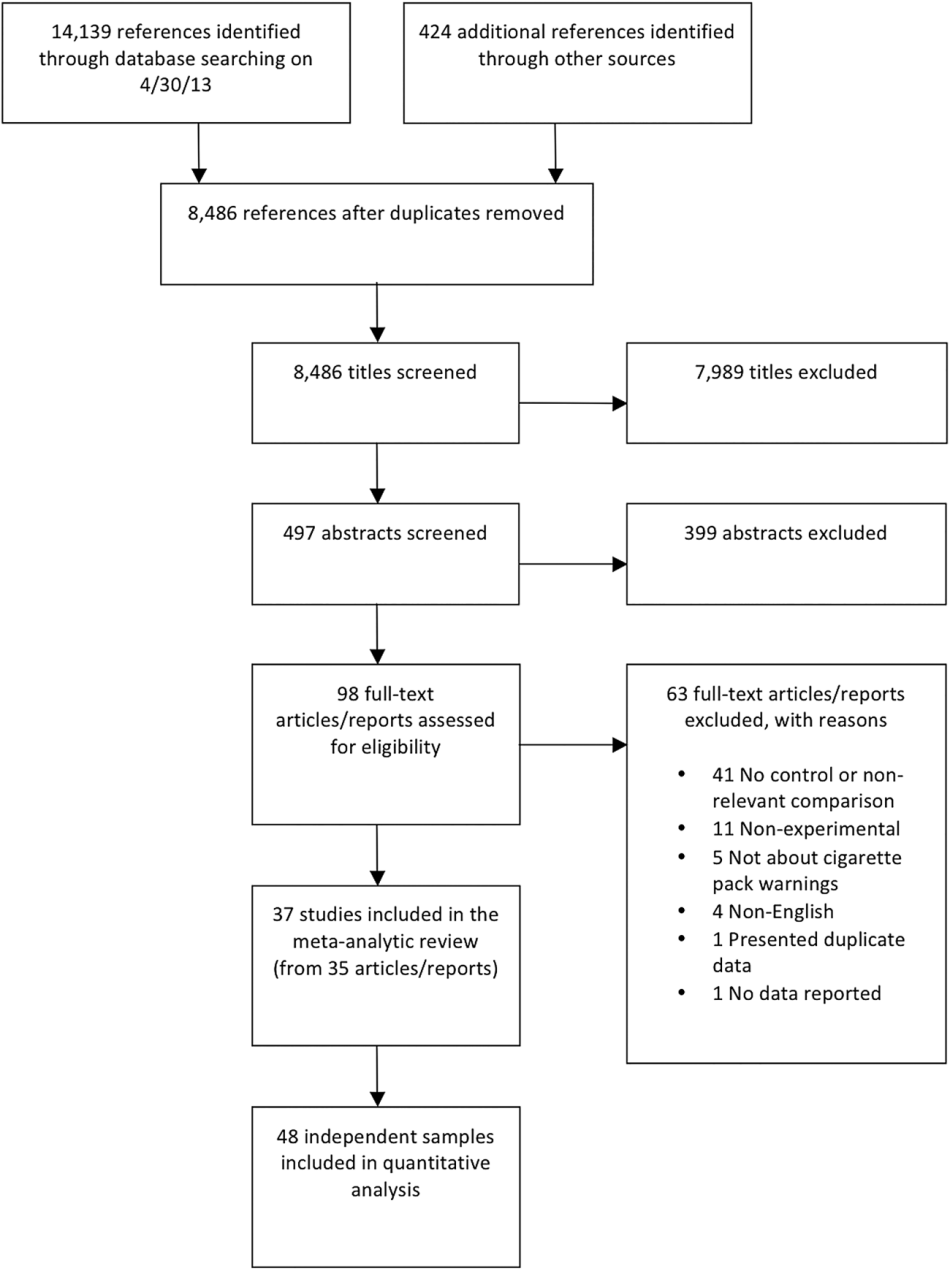
PRISMA flow diagram showing the study screening process.

Figure 1 depicts the search process. The initial database search yielded 14 139 total references, and searching through the other methods yielded 424 references. After removing duplicates, there were 8486 references. Two reviewers independently examined all study titles for relevance, reducing the number to 497, and then reviewed abstracts, further reducing the number to 98. During this process, we excluded articles only if both reviewers independently determined the article as irrelevant. Then, the two reviewers independently examined the full text of the 98 articles and tracked reasons for study exclusion. If the two reviewers made a different determination about the classification of a particular article, they consulted with a third referee to resolve the discrepancy and make a final determination. This process resulted in a total of 35 articles reporting on 37 studies. Since some studies reported results separately for different subgroups, we analysed effect sizes for each independent sample. Thus, the meta-analysis synthesised effects of 48 independent samples.

### Article coding

#### Coding study characteristics

Two independent coders coded all articles on several features, including *participant characteristics* such as gender, age, race/ethnicity and country of origin, and *study characteristics* such as within-subject/between-subject design and use of theory. The coders also coded *warning characteristics*: warning type (pictorial, text), nature of pictorial labels (image only, image with text), whether pictorial text and control text matched, number of different labels viewed, number of times viewing each label, number of exposure sessions, exposure medium (warning only, warning on two-dimensional pack, warning on three-dimensional pack), exposure channel (digital, printed or paper, cigarette pack), exposure control (researcher-controlled exposure, participant-controlled exposure), and label order (random, non-random).

The coders and the first author met to discuss each article after it was coded to compare the two coders’ work. All discrepancies between coders were resolved through discussion between the two coders and the first author. We calculated intercoder reliability for each characteristic. Most categories had perfect agreement, and the mean per cent agreement was 96%. Cohen's κ^34^ had a mean of 0.94.

#### Coding dependent variables

We developed a list of more than 30 dependent variables assessed in the studies based on an initial review of the literature. We then grouped these outcome variables into theory-based construct categories. Table 1 lists the constructs that at least two studies assessed, along with our definition of the construct, an example item from a study in the meta-analysis, and examples of the authors’ original terminology. We grouped all constructs into five categories. The first group (*attention and recall*) assessed participants’ attention to warnings and ability to recognise or recall the warnings. The second group (*warning reactions*) assessed participants’ cognitive, emotional and physiological reactions to warnings. The third group (*attitudes and beliefs*) assessed participants’ smoking or cigarette pack-related attitudes and beliefs. The fourth group (*intentions*) assessed participants’ intentions or willingness to act. Finally, the fifth group (*perceived effectiveness*) assessed participants’ perceptions of the effectiveness of warning messages.

**Table 1:** Outcomes assessed in experimental pictorial warning studies

Construct	Definition	Example item	Examples of authors’ terminology
Attention and recall			
Attention attracting	The extent to which the warning attracted or grabbed the participant's attention	The pack grabbed my attention^29^	Attract attention, salience
Attention duration	Amount of time participant spent viewing the warning label	NA (objective measure)	Looking time
Response time	The amount of time it took participant to complete questions or click forward after viewing the warning label	NA (objective measure)	Response latencies, response time
Recall/recognition of warning text	Whether participant could remember warning text following exposure	Try to recall what the warning information on the package stated and type it in the box below^28^	Recall, aided recall, correctly recalling warning statement
Warning reactions—cognitive, emotional and physiological			
Cognitive elaboration	The extent to which the participant thought about the warning's content (eg, the harms of smoking)	To what extent, if at all, do those health warnings make you think about the health risks?^35^	Think about health risks of smoking, think about harms
Negative affective reactions	Negative emotional reactions to the warning, such as fear or disgust	How afraid, worried, uncomfortable or disgusted participants felt after having seen each warning^36^	Negative affect, emotional reactions, evoked fear, fear intensity
Credibility	Perceptions of believability or truthfulness of the warning	The pack is believable^37^	Credibility, perceived credibility, believability
Lower psychological reactance	Lack of a negative reaction in response to a perceived threat to one's freedom	How irritated, angry, annoyed, and aggravated the warnings made the participant (reverse coded)^38^	State reactance, emotional reactions
Lower smoking cravings	The extent to which one does not crave a cigarette	I want a cigarette right now (reverse coded)^39^	Cravings to smoke, aversion to smoking
Aversiveness	The extent to which the warning was difficult to look at	The pack was difficult to look at^29^	Pack difficult to look at
Attitudes and beliefs			
Negative pack/brand attitudes	Negative evaluation of the cigarette pack or brand	Attitudes toward the package of cigarettes: unfavourable/favourable, negative/positive, and bad/good (reverse coded)^28^	Package attractiveness, package attitude, brand attitude
Negative smoking attitudes	Negative evaluation of smoking behaviour	Smoking helps people relax, smoking helps to reduce stress, smoking helps to keep weight down (reverse coded)^40^	Attitude toward cigarettes, smoking-related stereotypes
Perceived likelihood of harm	Beliefs that smoking cigarettes is likely to lead to health-related harms	Please evaluate your future risk of developing each of the following diseases: lung cancer, etc^41^	Risk of smoking-related diseases, smoking effects scale, perceived susceptibility, vulnerability
Self-efficacy to quit	Confidence in one’s ability to quit smoking	I do not need help from anyone to quit smoking^39^	Quit efficacy, self-efficacy
Intentions			
Lower willingness to pay	Prices assigned to cigarette packs with and without pictorial warnings	NA (monetary amount)	Perceived value for the pack
Intention to not start smoking	Likelihood of not starting smoking	Do you think that you will smoke a cigarette at anytime during the next year?^40^	Intent to smoke, intentions to start smoking
Intention to quit smoking	Likelihood of quitting smoking	How likely do you think it is that you will try to quit smoking within the next 30 days?^39^	Intention to quit, quit intentions
Perceived effectiveness of warning labels to…			
Motivate me/others to not start smoking	Perception of warning message's motivational value for participant/others not starting smoking	How effective label would be in convincing youth not to start smoking^42^	Motivation to remain abstinent, effectiveness rating—convincing youth not to start
Motivate me to cut down smoking	Perception of warning message's motivational value for participant cutting down on smoking	Indicate the chances that they would reduce the number of cigarettes smoked if the image they were viewing appeared on the cigarette or tobacco brand they normally purchased^42^	Foregoing a cigarette, reduce consumption
Motivate me to quit smoking	Perception of warning message's motivational value for participant quitting smoking	The information presented on this package would help me quit smoking^25^	Perceived intentions to quit, motivation to quit smoking, perceived impact on the decision to quit smoking
Motivate others to quit smoking	Perception of warning message's motivational value for others quitting smoking	How effective label would be in motivating smokers to quit^42^	Motivate smokers to quit, encourage other smokers to quit
Motivate me/others to not smoke (composite asked of smoker/non-smoker samples together)	Perception of warning message's motivational value to not smoke	Due to this warning, I would cut down/not start smoking. My smoking behaviour would be influenced by this warning^43^	Encourage others to quit/discourage others from starting, effectiveness evaluation
Be generally effective (typically single item)	Perception of the general effectiveness of the warning message (no referent, such as participant or others, provided)	Overall, on a scale of 1–10, how effective is this health warning?^44^	Overall effectiveness, most effective
Be effective for me/others (multiple-item scale)	Perceptions about the effectiveness of the warning message for participant /others	Multiple item scales, such as: the pack makes me want to quit smoking. The pack will make people more concerned about the health risks of smoking. The pack will prevent young people from starting to smoke^37^	Perceived effectiveness, perceived impact
Deter giving cigarettes as gift	Perceptions of the extent to which a warning label would deter a participant from wanting to give cigarettes as a gift	If you want to use cigarettes as a gift, do the following cigarette labels make you change your mind and not do so?^45^	Perceived impact of giving cigarettes as a gift

**Figure 2:**
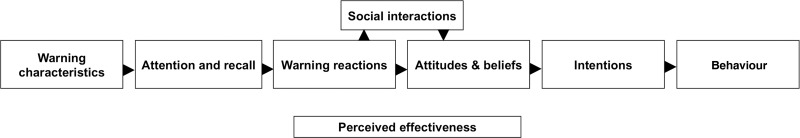
Message impact framework applied to research on cigarette pack warnings.

We organised these five groups of constructs into a message impact framework (figure 2), which is based on communication and psychological theory^46–52^ and previous tobacco warnings theory and research.^29^ ^53–55^ The framework suggests that the characteristics of a warning affect the extent to which the warning will be noticed and later recalled, and that attention to (and recall of) the warning influences warning reactions. Warning reactions are thought to, in turn, affect attitudes/beliefs, which later influence intentions and ultimately behaviour. Given that cigarette pack warnings are often in public view, they may spark interpersonal communication and social interactions.^49^ These social interactions, such as talking about the warnings with friends and family members, may affect individuals’ attitudes, beliefs, and reactions to the warnings.

Perceived effectiveness is also pictured in figure 2, and such ratings are commonly used in formative work to develop and assess messages.^56^ ^57^ However, currently, there is no evidence to suggest that these ratings play a direct role in warning message effects (ie, that participants must perceive a message to be effective in order for it to be so). Thus, perceived effectiveness is not pictured as an integral part of this framework.

### Effect-size extraction and calculation

We characterised the effect size of the benefit of pictorial over text warnings by using the standardised mean difference statistic d (ie, the difference in treatment and control means divided by the pooled SD).^58^ Because d can be upwardly biased when based on small sample sizes,^59^ we applied the recommended statistical correction for this bias.^58^ We calculated effect sizes from data reported in the article (eg, means and SDs; frequencies) using standard formulas.^58^ For within-subject designs, using statistics such as t and F for effect-size computation can bias effect-size estimates.^60^ However, using raw statistics such as means and SDs does not yield this bias.^60^ ^61^ Thus, we applied conventional formulas^58^ and computed all within-subject effect sizes from raw (vs inferential) statistics. If the article did not provide data necessary for effect-size computation, we requested the necessary data from authors.

We computed effect sizes for outcomes that were (1) identified as a meaningful construct from the communication or psychological literature and (2) assessed in two or more studies. When studies reported multiple pictorial warning or text-only conditions, we averaged these (text or pictorial) conditions together when computing effects. When studies reported more than one measure of the same variable (eg, two measures of negative smoking attitudes), we averaged them together. In order to keep effect sizes consistent and interpretable, we gave a positive sign (+) to effect sizes in which the pictorial warning condition performed better (ie, yielded a finding conducive to behavioural change) than the text-only condition, and a negative sign (−) to effect sizes in which the pictorial warning condition performed worse than the text-only condition.

### Meta-analytic approach

Analyses weighted effect sizes by their inverse variance and combined them using random effects meta-analytic procedures.^58^ We calculated the Q statistic and I^2^ to examine whether heterogeneity existed among the effect sizes. Most dependent variables had too few studies to perform moderator analyses. As some form of perceived effectiveness for *motivation to not smoke* was commonly assessed, we created a composite variable to use in moderator analyses. This composite variable consisted of all relevant perceived effectiveness motivation variables (ie, the first 5 constructs listed in table 4), and assessed the extent to which participants perceived pictorial warnings as motivating smokers or non-smokers to avoid smoking cigarettes. For the seven studies that measured a *motivation to avoid cigarette use* construct in multiple ways, we averaged together the effect sizes for all relevant outcomes. We performed moderator analyses on this variable using mixed-effects analyses, which allowed for the possibility of differing variances across subgroups.^58^ We calculated effect sizes for hypothesised categorical moderators along with their 95% CIs, and we statistically compared those effect sizes using the Q_b_ statistic. We also examined correlations between continuous moderator variables and effect size. We conducted all analyses using Comprehensive Meta-Analysis software V.2.2.046 and SPSS V.21.

## Results

### Study characteristics

The 37 studies were conducted in 16 different countries, with the most conducted in the USA (43%), followed by Canada (11%) and Germany (11%) (see online supplementary file).^23–25^ ^27–29^ ^35–45^ ^62–79^ While studies were published as early as 2000, most studies (68%) were published between 2009 and 2013. Fifty per cent of study samples included both smokers and non-smokers, 47% were smokers only, and one study was of non-smokers only. Most studies (65%) included both young adults and adults but not adolescents. Eleven studies (29%) included adolescents in their sample, although only four studies (11%) focused solely on adolescents. Study sample sizes ranged from 25 to 4890 (median=197), and the cumulative sample size across all studies was 33 613. Nineteen of 37 studies (51%) mentioned a theory as informing the study.

Studies varied considerably in how many different warnings they showed to participants (mean number of pictorial warnings=6.39, SD=10.86; mean number of text warnings=5.24, SD=10.91). However, in most studies, participants viewed a particular warning only once (86%), and they participated in only one viewing session (97%; table 2). In all but one study, participants were assessed only immediately after viewing the warning labels. The most commonly used exposure medium for warnings (57%) was a two-dimensional pack displayed on a computer with the participant controlling the duration of the exposure to the warning (ie, how long they viewed the warning before advancing further in the survey). Most pictorial warnings (89%) included both images and text, though some (8%) consisted of images only. In many cases (43%), the text in the pictorial warning matched the text presented in the comparison condition, though in several cases the text differed (43%).

**Table 2:** Characteristics of warning manipulations in studies in the meta-analysis

Variable	Pictorial (k=37)		Text (k=37)	
	k	Per cent	k	Per cent
Number of different warnings viewed				
1 warning	14	38	15	41
2–64 warnings	22	59	20	54
Not reported	1	3	2	5
Number of times viewed each warning				
1 time	32	86	32	86
2–5 times	5	14	5	14
Number of exposure sessions				
1 session	36	97	36	97
2–4 sessions	1	3	1	3
Days from exposure to assessment				
0 days (immediate assessment)	36	97	36	97
1–28 days	1	3	1	3
Exposure medium				
Just warning	4	11	6	16
Warning on a 2D pack	21	57	20	54
Warning on a 3D pack	8	22	8	22
Not reported	4	11	3	8
Exposure channel				
Digital	21	57	21	57
Printed or paper	4	11	4	11
Cigarette pack	8	22	8	22
Not reported	4	11	4	11
Label order				
Random	10	27	9	24
Non-random	5	14	5	14
Not reported	6	16	5	14
NA (1 label or all shown at once)	16	43	18	49
Warning exposure controlled by…				
Researcher	9	24	9	24
Participant	21	57	21	57
Both	1	3	1	3
Not reported	6	16	6	16
Nature of pictorial warnings				
Image only	3	8	–	–
Image with text	33	89	–	–
Not reported	1	3	–	–
Pictorial text vs comparison text				
Matched completely	16	43	–	–
Did not match	16	43	–	–
NA (pictorial condition had no text)	3	8	–	–
Not reported	2	6	–	–

All but a single study^69^ assessed individuals only directly after exposure.

2D, 2-dimensional; 3D, 3-dimensional; k, number of effect sizes; NA, not applicable.

Studies assessed more than 30 unique constructs (see online supplementary file). Each individual study assessed between one and eight constructs (M=2.75, SD=1.96). We identified 25 constructs that appeared in at least two studies, and these constructs are the focus of the meta-analysis (table 1).

### Effectiveness of pictorial warnings

**Figure 3:**
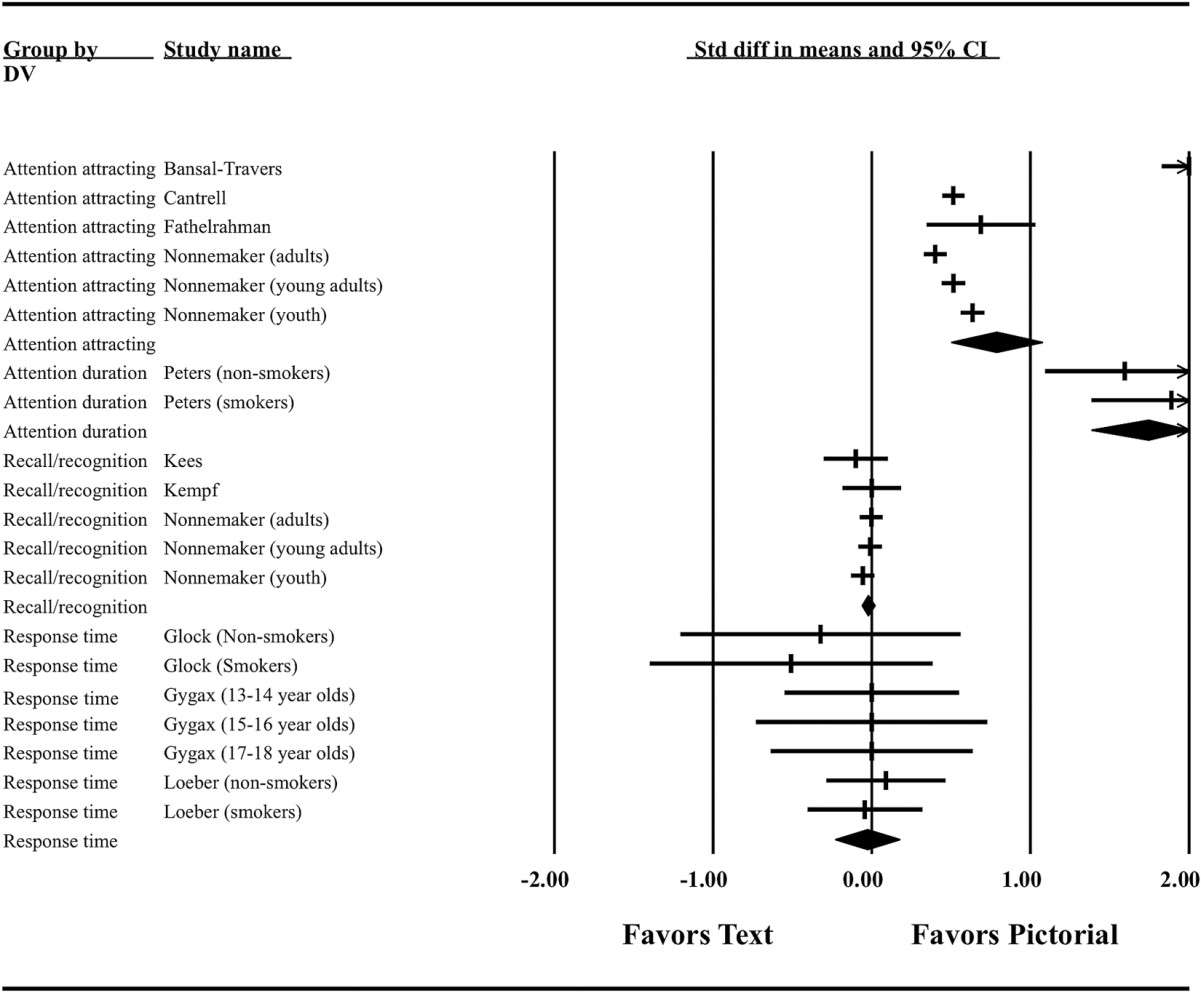
Forest plot displaying effect sizes and 95% CIs for attention outcomes.

Pictorial warnings exhibited statistically significant effects relative to text warnings for 13 of 17 effectiveness outcomes (most at p<0.001; see table 3), with 12 of 17 effects favouring pictorial warnings. Compared with text-only warnings, pictorial warnings showed an advantage for two of four *attention* constructs (figure 3), with pictorial warnings scoring higher on both attention attracting (d=0.79) and attention duration (d=1.74). We observed no effects on response time or recall/recognition of warning text.

**Table 3:** Effectiveness of pictorial warnings: mean weighted effect sizes (d) and heterogeneity statistics

	N	k	d	95% CI	p Value	Q	p Value	I^2^
Attention and recall								
Attention attracting	18 379	6	0.79	(0.50 to 1.07)	0.001	301	0.001	98
Attention duration	169	2	1.74	(1.39 to 2.10)	0.001	<1	0.42	0
Response time	386	7	−0.03	(−0.23 to 0.17)	0.77	2	0.92	0
Recall/recognition of warning text	15 052	5	−0.03	(−0.06 to 0.02)	0.22	2	0.76	0
Warning reactions—cognitive, emotional and physiological								
Cognitive elaboration	2082	3	1.70	(0.85 to 2.55)	0.001	105	0.001	98
Negative affective reactions	16 906	11	0.54	(0.44 to 0.64)	0.001	44	0.001	77
Credibility	20 222	9	0.15	(0.07 to 0.23)	0.001	35	0.001	77
Lower psychological reactance	14 324	4	−0.50	(−0.70 to −0.30)	0.001	61	0.001	95
Lower smoking cravings	3347	2	0.08	(0.01 to 0.16)	0.03	<1	0.68	0
Aversiveness	14 074	3	0.58	(0.42 to 0.75)	0.001	31	0.001	93
Attitudes/beliefs								
Negative pack/brand attitudes	1260	7	0.79	(0.50 to 1.07)	0.001	28	0.001	78
Negative smoking attitudes	489	4	0.55	(0.28 to 0.83)	0.001	6	0.11	51
Perceived likelihood of harm	14 460	8	0.02	(−0.04 to 0.07)	0.65	13	0.06	48
Self-efficacy to quit	3385	2	0.01	(−0.06 to 0.08)	0.80	<1	0.96	0
Intentions								
Lower willingness to pay	580	2	0.26	(0.02 to 0.50)	0.04	2	0.17	47
Intention to not start smoking	5016	4	1.82	(0.15 to 3.49)	0.03	336	0.001	99
Intention to quit smoking	16 671	8	0.54	(0.29 to 0.79)	0.001	256	0.001	97

n, number of participants; k, number of effect sizes; d, standardised mean difference (pooled effect size).

**Figure 4:**
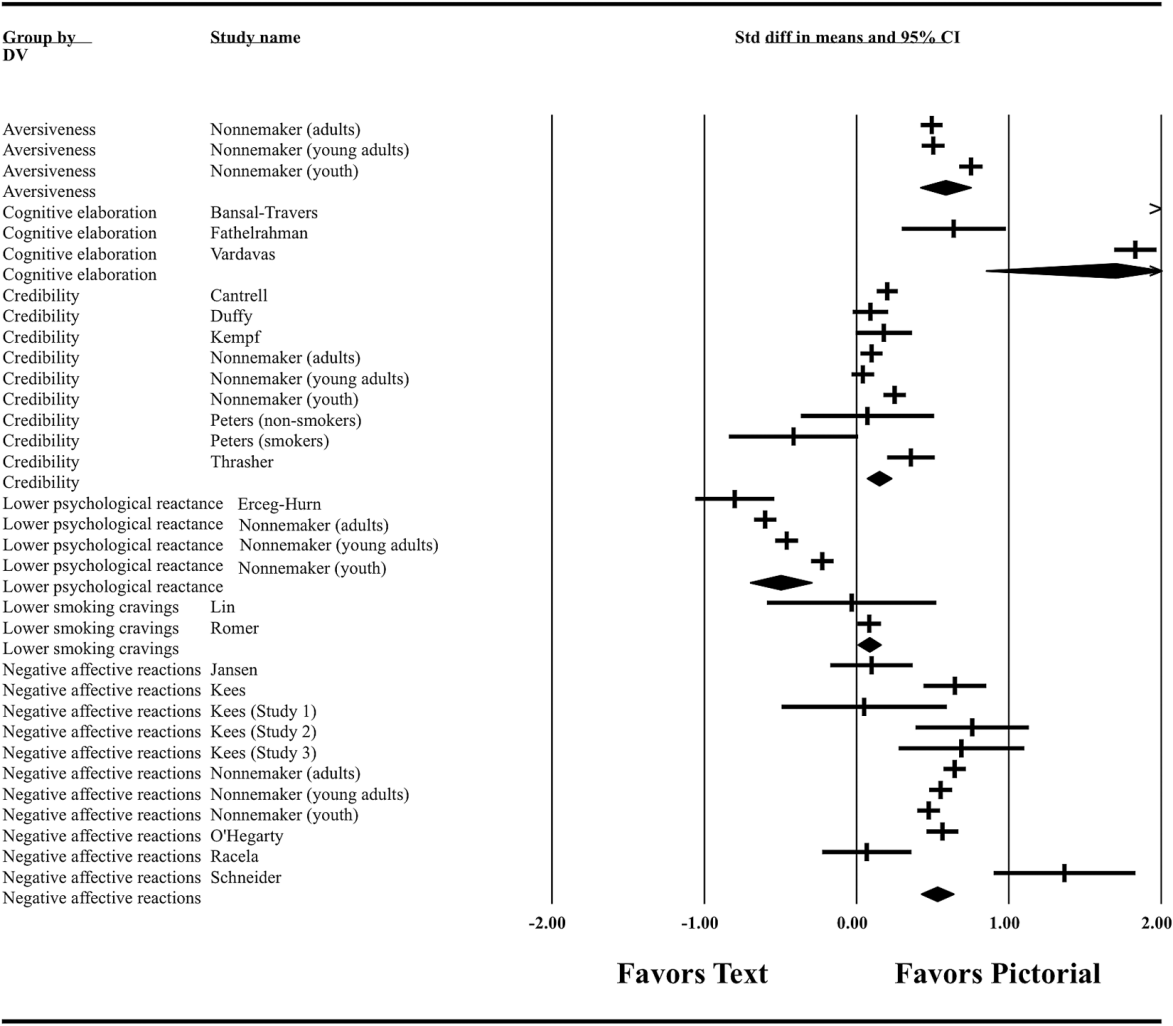
Forest plot displaying effect sizes and 95% CIs for warning reactions.

For *warning reactions*, pictorial warnings showed an advantage for five of six constructs (figure 4). Relative to text warnings, pictorial warnings elicited more cognitive elaboration (d=1.70), negative affective reactions (d=0.54), credibility (d=0.15), lower smoking cravings (d=0.08), and aversiveness (d=0.58). However, pictorial warnings also elicited greater psychological reactance (d=−0.50).

**Figure 5:**
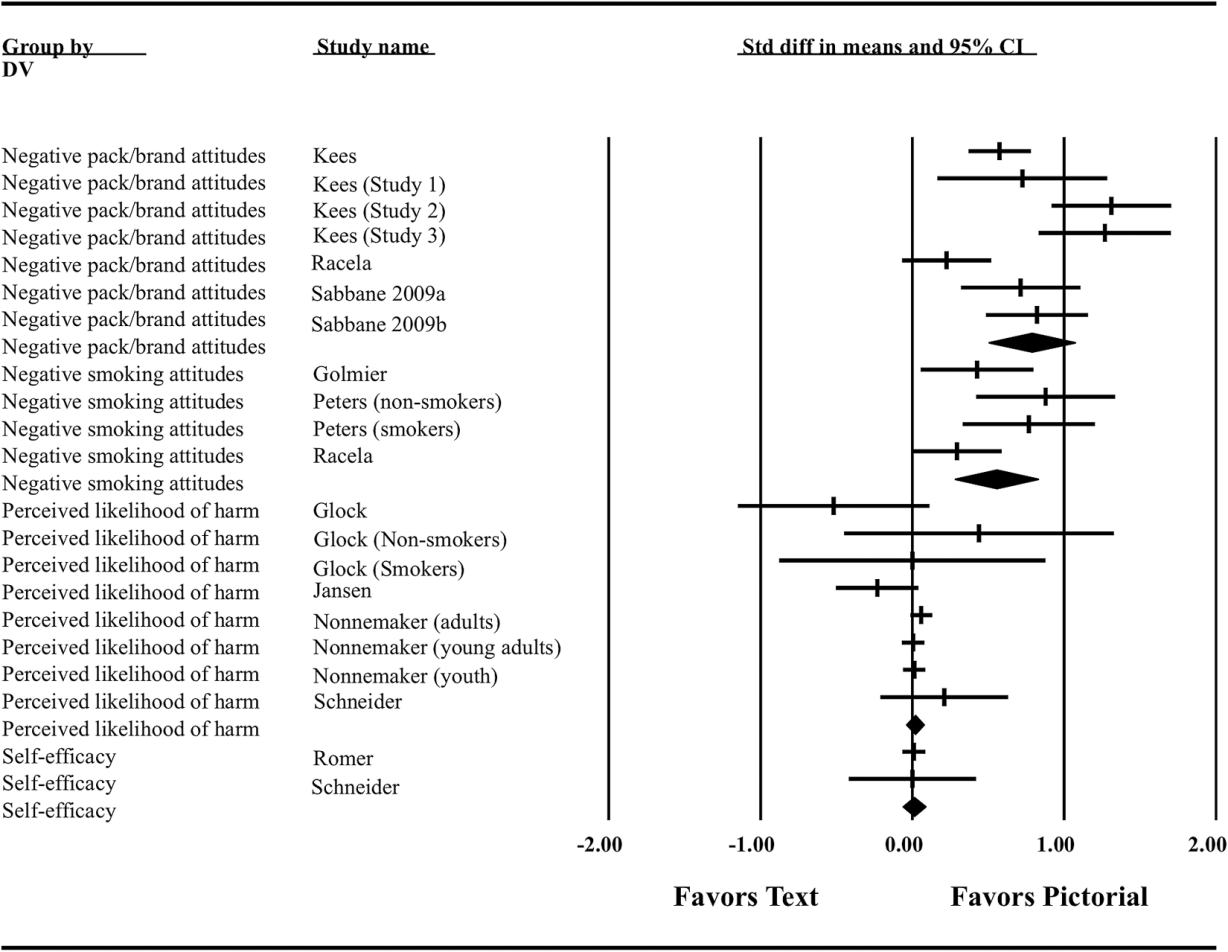
Forest plot displaying effect sizes and 95% CIs for attitudes/beliefs.

**Figure 6:**
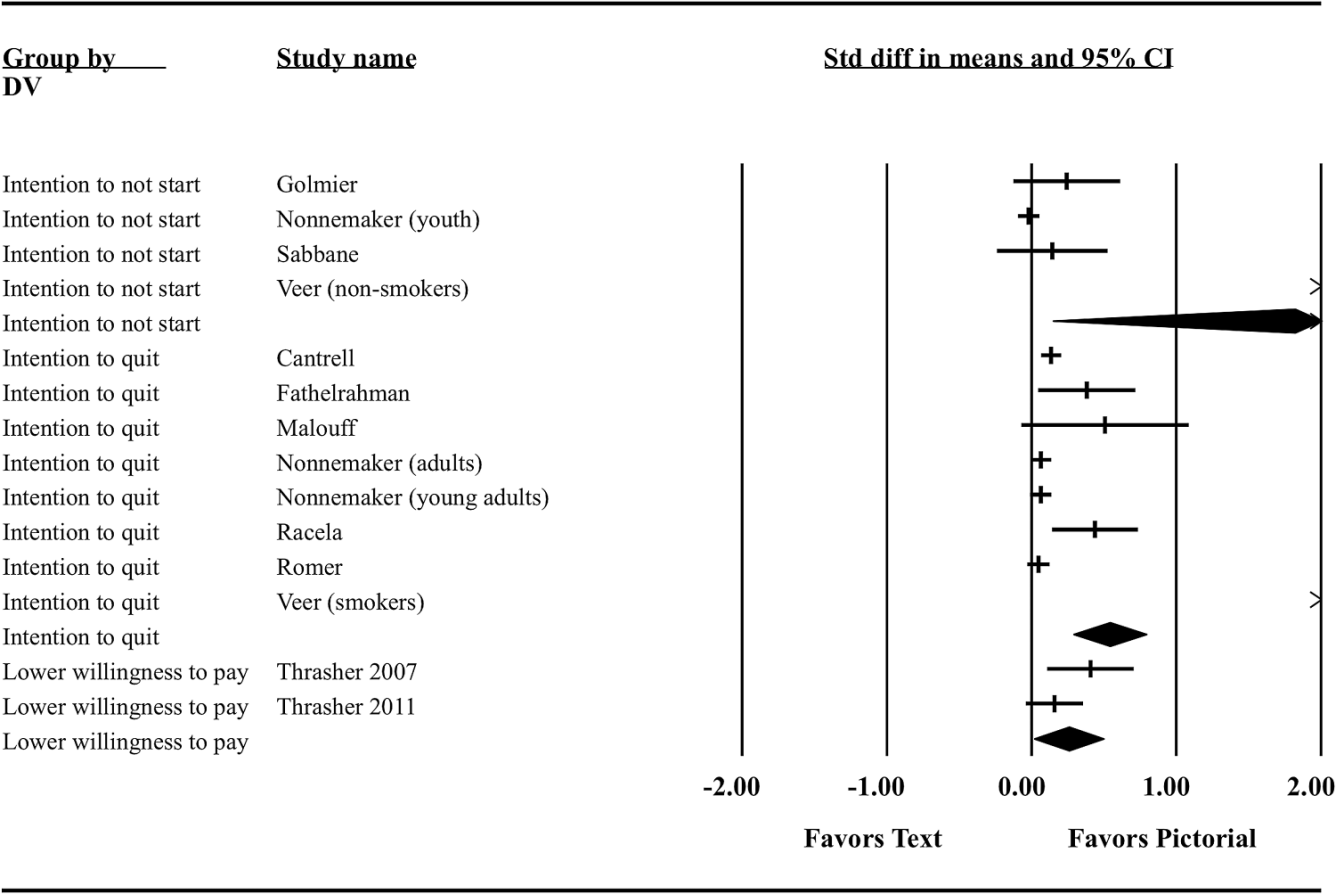
Forest plot displaying effect sizes and 95% CIs for intentions.

Pictorial warnings showed an advantage on two of four *attitude and belief* constructs (figure 5), with effects on both negative pack/brand attitudes (d=0.79) and negative smoking attitudes (d=0.55) relative to text warnings. No effects were observed on perceived likelihood of harm (d=0.02) or self-efficacy to quit (d=0.01). Moreover, pictorial warnings showed an advantage on all three *intentions* constructs (figure 6), with effects on lower willingness to pay (d=0.26), intention to not start smoking (d=1.82), and intention to quit smoking (d=0.54).

Homogeneity analyses indicated that 9 of 17 effect sizes were heterogeneous, with many outcomes exhibiting extremely high heterogeneity: 6 of these 9 outcomes had an I^2^ of greater than 90 (table 3).

### Perceived effectiveness of pictorial warnings

**Figure 7:**
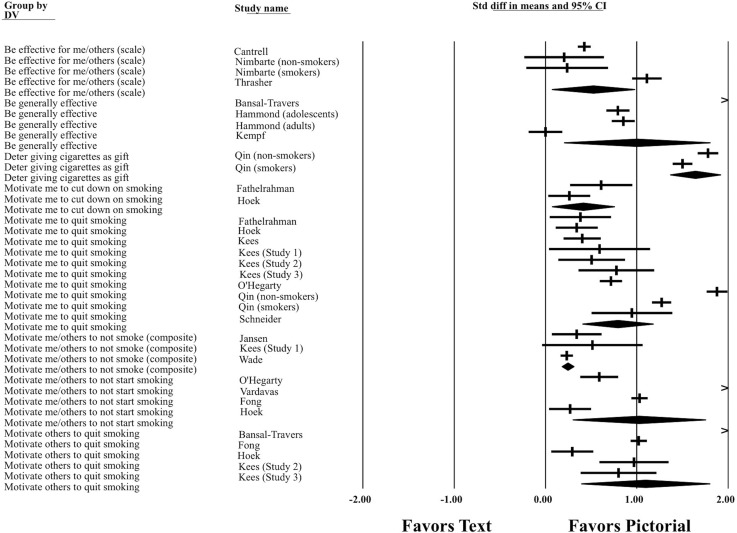
Forest plot displaying effect sizes and 95% CIs for perceived effectiveness.

Pictorial warnings exhibited statistically significant effects relative to text warnings for all eight *perceived effectiveness* outcomes (see table 4 and figure 7). Pictorial warnings were more likely to be rated as effective in motivating not starting smoking (d=1.03), motivating reducing smoking (d=0.41), motivating themselves (d=0.79) or others (d=1.09) to quit smoking, and motivating (smokers or non-smokers) to not smoke (d=0.24). Participants also perceived pictorial warnings as deterrents to giving cigarettes as a gift (d=1.64), as generally effective (d=1.00), and as effective for themselves and others (d=0.52).

**Table 4:** Perceived effectiveness of pictorial warnings: mean weighted effect sizes (d) and heterogeneity statistics

	N	k	d	95% CI	p Value	Q	p Value	I^2^
Perceived effectiveness of warning to…								
Motivate me/others to not start smoking	3946	4	1.03	(0.30 to 1.75)	0.006	251	0.001	99
Motivate me to cut down on smoking	450	2	0.41	(0.07 to 0.75)	0.02	3	0.09	64
Motivate me to quit smoking	5986	10	0.79	(0.41 to 1.18)	0.001	356	0.001	97
Motivate others to quit smoking	3667	5	1.09	(0.39 to 1.80)	0.002	238	0.001	98
Motivate me/others to not smoke (composite)	3807	3	0.24	(0.18 to 0.31)	0.001	2	0.47	0
Be generally effective (no referent)	3405	4	1.00	(0.20 to 1.80)	0.01	344	0.001	99
Be effective for me/others (scale)	4512	4	0.52	(0.07 to 0.97)	0.02	63	0.001	95
Deter giving cigarettes as gift	3504	2	1.64	(1.37 to 1.91)	0.001	13	0.001	92

n, number of participants; k, number of effect sizes; d, standardised mean difference (pooled effect size).

### Moderator analyses

The weighted mean effect size for the composite variable *motivation to avoid cigarette use* was statistically significant (p<0.001) at d=0.95 (CI 0.56 to 1.34, k=15, cumulative n=13 023). This effect was statistically heterogeneous, Q=1310, p<0.001, I^2^=99. Moderation analyses found that studies using a within-subject design (k=7) differed from those using a between-subject design (k=8; Q_b_=7.50, p<0.01; table 5). Studies using within-subject designs (d=1.37) had larger effect sizes than those using between-subject designs (d=0.51). Statistical comparisons of samples of smokers (k=9) to non-smokers and mixed samples (k=6) did not reach statistical significance (p=0.07). The trend, however, suggested that non-smokers and mixed samples (d=1.39), rated warnings as being more effective than did smokers (d=0.65). Analyses of exposure medium (two-dimensional vs three-dimensional pack), exposure channel (digital vs paper or pack), and country of sample (USA vs other countries) found no differences. Effect sizes were also not significantly correlated with gender composition, r (14)=−0.02 (p=0.98) or age, r(14)=−0.32 (p=0.49).

**Table 5:** Moderators of perceived motivation to avoid cigarette use

	k	d	95% CI	Q_b_ p Value
Study design				
Within subjects	7	1.37**	(0.78 to 1.97)	
Between subjects	8	0.51**	(0.36 to 0.66)	0.006
Participant smoking status				
Smokers	9	0.65**	(0.31 to 0.99)	
Non-smokers and mixed samples	6	1.39**	(0.67 to 2.11)	0.07
Country of sample				
USA	4	1.09*	(0.03 to 2.14)	
Other countries	11	0.90**	(0.47 to 1.34)	0.10
Exposure medium				
Warning on a 2D pack	10	1.02**	(0.56 to 1.47)	
Warning on a 3D pack	4	0.93	(−0.30 to 2.16)	0.90
Exposure channel				
Digital	5	0.96*	(0.14 to 1.78)	
Printed or paper	5	0.95**	(0.34 to 1.57)	
Cigarette pack	4	0.93	(−0.30 to 2.16)	0.99

*p<0.05, **p<0.001.

2D, 2-dimensional; 3D, 3-dimensional; k, number of studies; d, weighted mean effect size.

## Discussion

**Figure 8:**
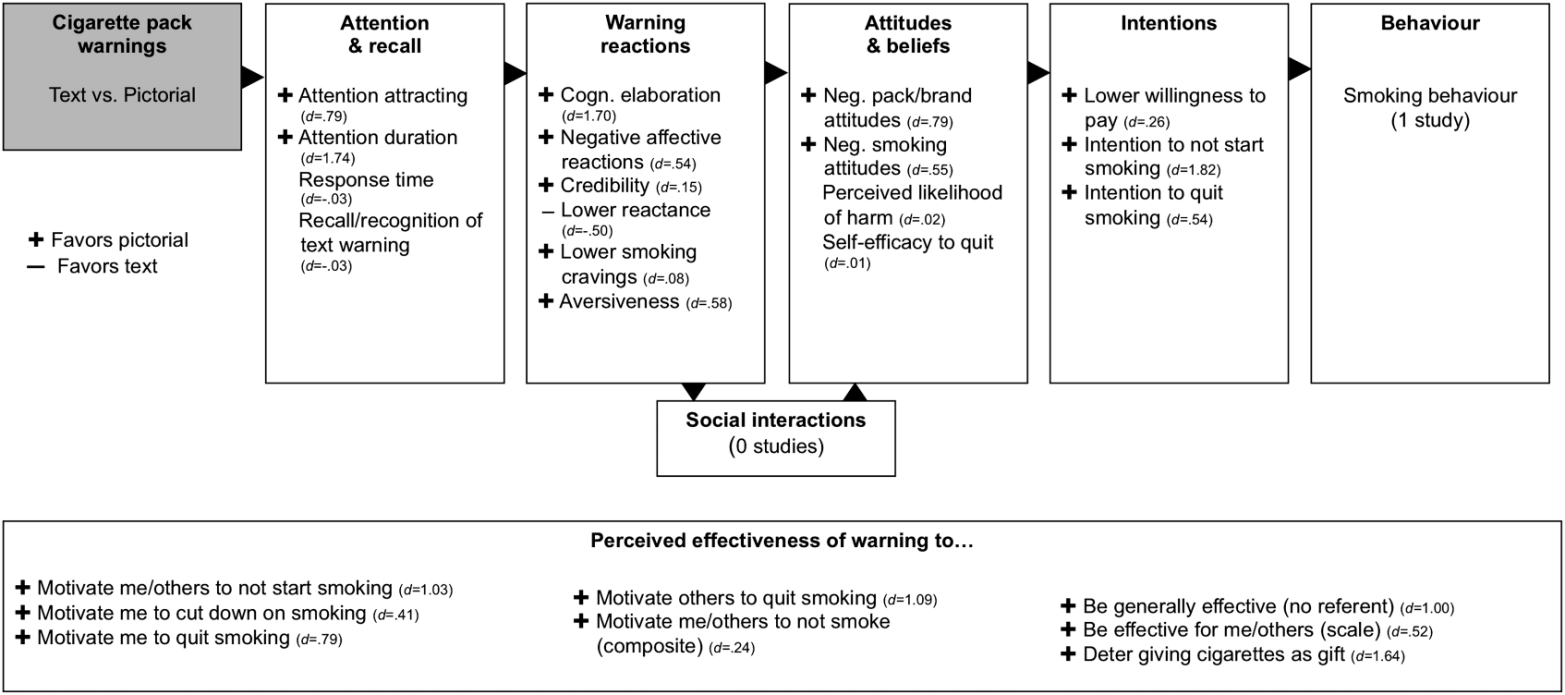
Effects of pictorial warnings on cigarette packs (summary of findings).

The purpose of this meta-analysis was to expand our understanding of the impact of pictorial cigarette pack warnings on smoking-related outcomes. Across an international body of experimental studies, we found effects favouring pictorial warnings for 12 of 17 effectiveness outcomes. Compared with text warnings, pictorial warnings (1) attracted and held attention better; (2) garnered stronger cognitive and emotional reactions; (3) elicited more negative pack attitudes and negative smoking attitudes; and (4) more effectively increased intentions to not start smoking and to quit smoking. These findings suggest that pictorial warnings are superior to text warnings at multiple stages of our message impact framework (figure 8) and may move people towards quitting smoking. While a recent systematic review did not find evidence that pictorial warnings were effective,^30^ that review examined only smoking behaviour and included mostly observational studies. The experimental studies we examined here showed promising evidence of effects.

In our meta-analysis, it is especially noteworthy that pictorial warnings changed negative smoking attitudes and quit intentions, as these variables are associated with quitting behaviour. For example, a previous meta-analysis of eight longitudinal studies found that negative smoking attitudes and quit intentions predicted subsequent quit attempts.^80^ Our review also demonstrated that pictorial warnings increased cognitive elaboration more than text-only warnings. Cognitive elaboration may play a particularly important role in the quitting process. A recent longitudinal study found that increased attention to cigarette pack warnings led to greater cognitive elaboration, which ultimately predicted quit attempts (via mediation through worry and quit intentions).^81^

In our review, pictorial warnings were also superior to text warnings on all eight perceived effectiveness outcomes. Smokers and non-smokers rated pictorial warnings as more effective than text warnings at motivating not starting, reducing and quitting smoking. These findings are noteworthy, as research has suggested that messages with higher perceived effectiveness ratings may be more effective than those with lower ratings.^82–84^ Taken together, these findings on effectiveness (eg, increased quit intentions) and perceived effectiveness (eg, increased perception that warnings motivate quitting) offer strong evidence to support pictorial cigarette pack warnings as more effective than text-only warnings.

### Mediators of pictorial warning effects

Our meta-analysis provides support for the notion that pictorial warnings elicit changes in an array of psychosocial constructs that are plausible mediators of the warning-behaviour link. Future studies should identify constructs that mediate pictorial warnings’ effects on smoking behaviour. The potential mediator most proximal to behaviour is intentions, one of the strongest predictors of behaviour according to both theory^85^ and empirical research.^80^

We saw the effects for many beliefs and attitudes that are plausible mediators according to theories of health behaviour.^50^ ^85^ ^86^ In our meta-analysis, pictorial warnings elicited greater fear-oriented reactions than text warnings, as intended. This is consistent with previous research and theory on fear appeals, which has found that such appeals increase fear as a mechanism for attitude, intention and behavioural change.^87^ However, fear appeal theories, such as the extended parallel process model (EPPM),^52^ also suggest that two key constructs help explain how people respond to fear appeals—perceived threat and efficacy. On these two key constructs, we found no effects of pictorial warnings. It was surprising that only five studies (with 8 effect sizes) in the meta-analysis assessed perceived likelihood of harm, a component of perceived threat, when much theorising situates this as a central construct in fear appeals and risk communication.^52^ ^88^ ^89^ While our meta-analysis did not find an effect of pictorial warnings on perceived likelihood of harm, the reason is unclear. It may be due to inadequate perceived likelihood measures or a failure to change risk beliefs because of an inadequate dose of warning exposure. It is also important to note that the lone study that assessed perceived *severity* of harm, another component of perceived threat, found a large effect.^36^ More careful studies of the impact of pictorial warnings on perceived likelihood and severity are required before we can make stronger conclusions regarding the role of risk beliefs in warning effectiveness.

Moreover, considering the potential importance of self-efficacy in predicting how people respond to health messages, pictorial warnings may be more effective if they increase self-efficacy to quit smoking. However, only two studies in this meta-analysis measured self-efficacy, and none manipulated it experimentally. Several countries, including Brazil, Australia and New Zealand, require that pictorial warnings provide information about cessation services, which may be a promising strategy for increasing smokers’ self-efficacy to quit.^90^ ^91^ Future pictorial warning studies should examine the role of self-efficacy in predicting changes in intentions and behaviour, and the interaction of efficacy and threat, testing hypotheses from the EPPM.^52^

Previous fear appeal research and theory also suggest that fear-oriented communications can elicit reactance.^87^ While we found that pictorial warnings elicited greater reactance than text warnings, the studies in our meta-analysis focused on the emotional aspect of reactance, and largely ignored the cognitive elements. Research characterises reactance as a construct comprised of both emotion (eg, anger, irritation) and cognition (eg, defensive processing, denial).^92^ Future studies on pictorial warnings should advance a more comprehensive measurement approach and should examine whether reactance leads to adverse outcomes, such as lower quit intentions or greater smoking behaviour.

Finally, our meta-analysis found pictorial warnings increased aversiveness (ie, the warnings being ‘difficult to look at’). How aversiveness plays into effects of such warnings is unclear. For example, actions such as looking away from, covering up, or avoiding the warnings may reduce the warnings’ effects; alternatively, these behaviours may actually be markers of the warnings’ effectiveness. One observational study revealed that Canadian smokers who avoided pictorial warnings were equally likely to think about the warnings or engage in cessation behaviour than those who did not attempt to avoid the warnings.^93^ Further research is needed to understand how aversiveness affects pictorial warning effectiveness.

### Theoretical and measurement issues

Despite the existence of several models to guide warnings research,^53–55^ our meta-analysis revealed a lack of consensus as to what outcomes experimental studies should assess. Outcomes varied widely, with little consistency across studies. The framework presented in this article (figure 2), along with our empirical findings (figure 8), may help bring theoretical clarity to the literature. In particular, we recommend that researchers pay particular attention to issues of construct validity, taking care to explicitly describe what they measure and how they measure it, and ensuring that the name accurately matches what the measure is assessing. Researchers should also carefully consider what types of constructs and measures are most appropriate for their study given the stage of warnings research in a given country, and the goals of the particular study.

### Study design

Our meta-analysis included both experiments that manipulated pictorial warnings between subjects (participants viewed only text or only pictorial warnings) and within subjects (participants viewed both warning types). The between-subject studies had markedly smaller effect sizes for *perceived effectiveness for motivation to avoid cigarette use* than within-subject studies. Why was this the case? One explanation may be a reference point effect.^94^ That is, when one evaluates a text warning followed by a pictorial warning, the pictorial warning seems that much more powerful. In that case, the participant may rate the pictorial warning higher than would have been the case otherwise.^95^ Another possibility is that seeing multiple warnings makes it easier to focus on the presence or absence of graphics, the central attribute that differs. This is similar to Hsee's evaluability hypothesis that explains what happens when an important attribute is hard to evaluate independently.^96^ In the real world, participants are likely to see only text *or* pictorial warnings on cigarette packs, and so it may be that between-subject studies provide a more accurate estimate of effect size of that difference. However, comparisons among different pictorial warnings may best be done using within-subject studies, as people will likely see many such warnings on packs.

A final study design issue is that no study in this meta-analysis tested warnings by placing them on smokers’ cigarette packs. Instead, participants had only brief exposure to warnings, often on a computer screen. By contrast, the large body of observational literature examines smokers who have had multiple exposures to warnings on their cigarette packs.^8^ Such studies are invaluable, as they can potentially demonstrate population-level effects that result from warning policy changes. However, internal validity threats make strong causal conclusions from such studies difficult, especially on outcomes such as smoking behaviour.^30^ Therefore, we recommend that future experimental studies place warnings on smokers’ cigarette packs, follow participants over time, and assess smoking behaviour as an outcome.^97–99^

### Gaps and future directions

This review identified several areas for future research. First, our meta-analysis identified only a single experimental study that assessed behaviour.^69^ Future experimental pictorial warning studies should place warnings on smokers’ cigarette packs, measure smoking behaviour over longer periods of time, and include meaningfully intensive exposures.^97^ Second, given that smoking is a social behaviour,^100^ a better understanding of how social interactions influence warning effectiveness is needed. While some studies have assessed discussions about warnings,^19^ ^93^ ^101^ ^102^ studies often use this variable in a composite scale representing depth of processing, making it impossible to tease out the influence of social interactions. Third and finally, an area that remains understudied is the effect of pictorial warnings on reducing smoking initiation. While warnings may be seen as messages designed only for smokers, some warnings could be specifically designed for youth and non-smokers.^19^ Future research should examine the differential impact of pictorial warnings on smokers and non-smokers, with careful attention to the potential for pictorial warnings to discourage smoking initiation among youth.

## Conclusion

Our study was the first to estimate the effects of pictorial cigarette pack warnings through a meta-analysis of experimental studies. This investigation demonstrated that pictorial warnings were more effective than text warnings on the vast majority of outcomes studied, affecting several constructs, including intention to not start smoking and intention to quit smoking. Future research examining the effects of pictorial cigarette warnings should assess impact on smoking behaviour, including initiation and cessation. Studies should also adopt more explicit hypotheses derived from behavioural theory, use validated and standardised measures, include multiple follow-up assessments, and better advance a theoretical understanding of how warnings exert their effects.

**What this paper adds**

- Pictorial warnings on cigarette packs are a key international tobacco control policy. The current study presents the first meta-analysis of the experimental literature on pictorial cigarette pack warnings. This review found:
  Pictorial warnings were more effective than text warnings on 20 of 25 outcomes examined in the meta-analysis;
  Pictorial warnings were more effective (eg, increased quit intentions) and perceived to be more effective (eg, rated as likely to motivate smokers to quit) as compared with text warnings;
  Pictorial warnings were more effective than text warnings in changing outcomes relevant to both non-smokers (eg, intentions to not start smoking) and smokers (eg, intentions to quit smoking).
- Future experimental research should examine the impact of pictorial warnings on smoking behaviour. Future studies should also apply more behavioural theory, and test which theoretical variables mediate the effects of pictorial warnings. Such work would further strengthen the international evidence base for pictorial warnings and advance our ability to better understand the ‘active ingredients’ that underlie such warnings, informing more effective tobacco control policies.

## Supplementary Material

Web table
