# Supplementary material for: Pictorial cigarette pack warnings: a meta-analysis of experimental studies
Source: Tob Control. 2015 May 6;25(3):341–54. doi: 10.1136/tobaccocontrol-2014-051978 (PMC4636492; doi:10.1136/tobaccocontrol-2014-051978)
Supplement: Web table [file tobaccocontrol-2014-051978-s1.pdf]

Online Supplement. Study Characteristics of the 48 Independent Samples Included in the Meta-Analysis

Study	N	Age Groups	Mean age	% male	Country	Smoking Status	Probability Sample	Study Design	Theory	Dependent Variables and Effect Size (d)
Bansel-Travers (2011)[24]	397	YA, A	–	51	United States	Smokers and non-smokers	No	Within	–	Attention attracting (d=2.00)<br>Be generally effective (d=2.36)<br>Cognitive elaboration (d=2.59)<br>Lower purchase interest (d= 1.79)<br>Motivate others to quit smoking (d=2.36)
Cantrell (2013)[37]	3371	YA, A	44	39	United States	Smokers	Partial	Between	Knowledge gap, communication theory	Attention attracting (d=.51)<br>Be effective (scale) (d=.43)<br>Credibility (d=.20)<br>Intention to quit smoking (d=.14)
Duffy (2000)[62]	580	C, AD	–	43	United States	Smokers and non-smokers	No	Within	–	Credibility (d=.09)<br>Other – importance (d=.05)
Erceg-Hurn (2011)[38]	250	YA, A	29	54	Australia	Smokers	No	Between	Psychological reactance	Lower psychological reactance (d=-.80)
Fathelrahman (2010)[35]	140	YA, A	–	100	Malaysia	Smokers	No	Between	–	Attention attracting (d=.69)<br>Avoidance behavior (d=.18)<br>Cognitive elaboration (d=.64)<br>Intention to quit smoking (d=.38)<br>Knowledge (d=.50)<br>Motivate me to cut down on smoking (d=.61)<br>Motivate me to quit smoking (d=.38)
Fong (2010)[13]	1169	AD, YA, A	–	50	China	Smokers and non-smokers	No	Within	–	Motivate me/others to not start smoking (d=1.03)<br>Motivate others to quit smoking (d=1.02)
Glock (2009) - Smokers[41]	30	YA	22.4	7	Germany	Smokers	No	Between	Cognitive dissonance	Perceived likelihood of harm (d=.00)

									theory	Response time (d=-.51)
Glock (2009) - Non-Smokers[41]	30	YA	21.7	13	Germany	Non-smokers	No	Between	Cognitive dissonance theory	Perceived likelihood of harm (d=0.44)<br>Response time (d=-.32)
Glock (2012)[63]	60	YA, A	23	23	Germany	Smokers	No	Between	Fear appeals	Perceived likelihood of harm (d=-.52)
Golmier (2007)[40]	186	AD	14	56	Canada	Smokers and non-smokers	No	Between	Stereotype priming model	Intentions to not start smoking (d=.24)<br>Negative smoking attitudes (d=.43)
Gygax (2010) - 13-14 year olds[64]	51	AD	13.3	55	Switzer-land	Smokers and non-smokers	No	Between	Health belief model, Fear Appeals, Prospect Theory	Response time (d=.00)
Gygax (2010) - 15-16 year olds[64]	29	AD	15.2	48	Switzer-land	Smokers and non-smokers	No	Between	Health belief model, Fear appeals, Prospect theory	Response time (d=.00)
Gygax (2010) - 17-18 year olds[64]	38	AD	17.7	45	Switzer-land	Smokers and non-smokers	No	Between	Health belief model, Fear appeals, Prospect theory	Response time (d=.00)
Hammond (2012) - Adults[44]	544	YA, A	29.3	52	Mexico	Smokers	Yes	Within	Fear appeals	Be generally effective
Hammond (2012) - Adolescents[44]	528	AD	17	50	Mexico	Smokers and non-smokers	Yes	Within	Fear appeals	Be generally effective (d=.79)
Hoek (2006)[65]	310	YA, A	30	34	New Zealand	Smokers	No	Between	–	Motivate me to cut down on smoking (d=.26)<br>Motivate me to quit smoking (d=.32)

										Motivate me/others to not start smoking (d=.27)<br>Motivate others to quit smoking (d=.29)<br>Quitline (d=.19)
Jansen (2006)[43]	213	YA, A	21.3	43	Nether-lands, Belgium	Smokers	No	Between	Extended parallel process model, Fear appeals	Perceived likelihood of harm (d=-.23)<br>Motivate me/others to not smoke - composite (d=.34)<br>Negative affective reactions (d=.10)<br>Other – fear control mode (d=.65)
Kees (2006) - Study 1[25]	76	YA, A	22	59	United States	Smokers	No	Between	–	Motivate me to quit smoking (d=.59)<br>Motivate me/others to not smoke - composite (d=.51)<br>Negative affective reactions (d=.05)<br>Negative pack/brand attitudes (d=.73)
Kees (2006) - Study 2[25]	199	YA, A	–	0	United States	Smokers	–	Between	–	Motivate me to quit smoking (d=.51)<br>Motivate others to quit smoking (d=.97)<br>Negative affective reactions (d=.76)<br>Negative pack/brand attitudes (d=1.31)
Kees (2006) - Study 3[25]	145	YA, A	–	0	Canada	Smokers	–	Between	–	Motivate me to quit smoking (d=.78)<br>Motivate others to quit smoking (d=.80)<br>Negative affective reactions (d=.79)<br>Negative pack/brand attitudes (d=1.27)

Kees (2010)[28]	511	YA, A	48	–	United States, Canada	Smokers	–	Between	Fear appeals	Motivate me to quit smoking (d=.40)<br>Negative affective reactions (d=.65)<br>Negative pack/brand attitudes (d=.58)<br>Recall/recognition of warning text (d=-.10)
Kempf (2006)[66]	467	YA, A	22	54	United States	Smokers and non-smokers	No	Between	Fear appeals	Be generally effective (d=.00)<br>Credibility (d=.18)<br>Other – depth of processing (d=.00)<br>Recall/recognition of warning text (d=.00)
Lin (2011)[67]	25	YA, A	49	52	United States	Smokers	No	Within	–	Lower smoking cravings (d=.03)
Loeber (2011) - Non-Smokers[68]	55	YA, A	31	36	Germany	Non-smokers	Yes	Within	Attentional bias	Response Time (d=.09)
Loeber (2011) - Smokers[68]	59	YA, A	34.3	47	Germany	Smokers	Yes	Within	Attentional bias	Response Time (d=-.04)
Malouff (2012)[69]	56	YA, A	25.8	59	Australia	Smokers	No	Between	–	Intention to quit smoking (d=.51)<br>Smoking behavior (d=.16)
Nimbarte (2005) - Non-Smokers[70]	41	–	–	–	United States	Non-smokers	–	Within	–	Be effective (scale) (d=.20)
Nimbarte (2005) - Smokers[70]	39	–	–	–	United States	Smokers	–	Within	–	Be effective (scale) (d=.24)
Nonnemaker (2010) - Adults[29]	4,890	A	43.5	49	United States	Smokers	No	Between	Theory of reasoned action, Various message processing and health behavior	Attention attracting (d=.40)<br>Aversiveness (d=.49)<br>Perceived likelihood of harm (d=.06)<br>Credibility (d=.10)<br>Intention to quit smoking (d=.06)<br>Negative affective reactions

									theories	(d=.64)<br>Lower psychological reactance (d=-.60)<br>Recall/recognition of warning text (d=.00)
Nonnemaker (2010) - Young Adults[29]	4,584	YA	21.6	54	United States	Smokers	No	Between	Theory of reasoned action, Various message processing and health behavior theories	Attention attracting (d=.52)<br>Aversiveness (d=.50)<br>Perceived likelihood of harm (d=.01)<br>Credibility (d=.04)<br>Intention to quit smoking (d=.06)<br>Negative affective reactions (d=.55)<br>Lower psychological reactance (d=-.46)<br>Recall/recognition of warning text (d=-.01)
Nonnemaker (2010) - Youth[29]	4,600	AD	15.7	53	United States	Smokers and non-smokers	No	Between	Theory of reasoned action, Various message processing and health behavior theories	Attention attracting (d=.64)<br>Aversiveness (d=.75)<br>Perceived likelihood of harm (d=.01)<br>Credibility (d=.25)<br>Intention to not start smoking (d=-.02)<br>Negative affective reactions (d=.47)<br>Lower psychological reactance (d=-.23)<br>Recall/recognition of warning text (d=-.06)
O'Hegarty (2006)[23]	763	YA	–	43	United States	Smokers and non-smokers	Yes	Within	–	Motivate me to quit smoking (d=.72)<br>Motivate me/others not start smoking (d=.59)<br>Negative affective reactions (d=.56)

Peters (2007) – Non-Smokers[71]	81	YA, A	34	46	United States	Non-smokers	No	Between	Fear appeals, Defensive avoidance	Attention duration (d=1.59)<br>Credibility (d=.07)<br>Negative smoking attitudes (d=.88)
Peters (2007) – Smokers[71]	88	YA, A	37	70	United States	Smokers	No	Between	Fear appeals, Defensive avoidance	Attention duration (d=1.89)<br>Credibility (d=-.41)<br>Negative smoking attitudes (d=.77)
Qin (2011) – Non-Smokers[45]	714	YA, A	34	31	China	Non-smokers	No	Within	–	Deters giving cigarettes as gift (d=1.78)<br>Motivate me to quit smoking (d=1.88)<br>Other - Clarity
Qin (2011) – Smokers[45]	162	YA, A	34	94	China	Smokers	No	Within	–	Deter giving cigarettes as gift (d=1.50)<br>Motivate me to quit smoking (d=1.27)<br>Other - Clarity
Racela (2012)[72]	205	YA, A	–	83	Thailand	Smokers	Yes	Between	Fear appeals	Intention to quit smoking (d=.44)<br>Negative affective reactions (d=.07)<br>Negative pack/brand attitudes (d=.23)<br>Negative smoking attitudes (d=.29)<br>Perceived ethicality (d=.38)
Romer (2013)[39]	3297	YA, A	33.2	–	United States	Smokers	No	Between	Efficacy-desire model	Intention to quit smoking (d=.05)<br>Lower smoking cravings (d=-.08)<br>Self-efficacy (d=.01)
Sabbane (2009a)[73]	168	AD	–	47	Canada	Smokers and non-smokers	No	Between	General priming theory	Intention to not start smoking (d=.14)<br>Negative pack/brand attitudes (d=.71)
Sabbane (2009b)[27]	220	AD, YA	–	41	United States,	Non-smokers	No	Between	–	Negative pack/brand attitudes (d=.82)

					Canada					
Schneider (2012)[36]	88	YA, A	22	56	Germany	Smokers	No	Between	Extended parallel process model, Fear appeals, Protection motivation theory	Perceived likelihood of harm (d=.21)<br>Perceived severity of harm (d=.80)<br>Response efficacy (d=.51)<br>Motivate me to quit smoking (d=.95)<br>Negative affective reactions (d=1.37)<br>Self-efficacy (d=.00)
Thrasher (2007)[75]	89	YA, A	27.6	54	Mexico	Smokers	No	Within	–	Lower willingness to pay (d=.41)
Thrasher (2011)[76]	402	YA, A	38	56	United States	Smokers	No	Within	–	Lower willingness to pay (d=.16)
Thrasher (2012)[74]	981	YA, A	–	41	United States	Smokers	No	Between	–	Be effective (scale) (d=1.11)<br>Credibility (d=.36)<br>Personal relevance (d=.90)
Vardavas (2009)[77]	574	AD	15	46	Greece	Smokers and non-smokers	No	Within	–	Cognitive elaboration (d=1.83)<br>Motivate me/others to not start smoking (d=2.20)
Veer (2012) - Smokers[78]	194	YA, A	24	47	England	Smokers	No	Between	Terror management theory, Morality salience hypothesis	Intention to not start smoking (d=8.83)<br>Other – cognitive processing (d=13.87)
Veer (2012) - Non-Smokers[78]	136	YA, A	24	47	England	Non-smokers	No	Between	Terror management theory, Morality salience hypothesis	Intention to not start smoking (d=7.14)<br>Other - cognitive processing (d=4.28)
Wade	1778	AD,	–	–	Russia	Smokers	Yes	Within	–	Motivate me/others to not smoke -

(2010)[79]		YA, A				and non-smokers				composite (d=.23)

Note. C=children (ages 10 and under), AD=adolescents (ages 11-17), YA=young adults (ages 18-25), A=Adults (ages 26+), dash (–) = not reported. *N*=sample size; *d*=standardized mean difference (pooled effect size). Numbers refer to the references as listed in the main article.
